# Left ventricular septal pacing combined with left ventricular pacing improves acute electric resynchronization, hemodynamic responses and clinical outcomes: results of SPORT study

**DOI:** 10.1093/europace/euaf147

**Published:** 2025-08-04

**Authors:** Siyuan Xue, Chen He, Fengwei Zou, Jiaxin Zeng, Shun Xu, Yao Wang, Zhiyong Qian, Xinwei Zhang, Xiaofeng Hou, Xiaohan Fan, Jiangang Zou

**Affiliations:** Department of Cardiology, The First Affiliated Hospital, Nanjing Medical University, No. 300 Guangzhou Road, Nanjing 210029, China; Cardiac Arrhythmia Center, Fuwai Hospital, National Center for Cardiovascular Diseases, Chinese Academy of Medical Sciences and Peking Union Medical College, No. 167 Beilishi Road, Beijing 100037, China; Department of Cardiology, Montefiore Medical Center, Bronx, New York, USA; Department of Cardiology, The First Affiliated Hospital, Nanjing Medical University, No. 300 Guangzhou Road, Nanjing 210029, China; Department of Cardiology, The First Affiliated Hospital, Nanjing Medical University, No. 300 Guangzhou Road, Nanjing 210029, China; Department of Cardiology, The First Affiliated Hospital, Nanjing Medical University, No. 300 Guangzhou Road, Nanjing 210029, China; Department of Cardiology, The First Affiliated Hospital, Nanjing Medical University, No. 300 Guangzhou Road, Nanjing 210029, China; Department of Cardiology, The First Affiliated Hospital, Nanjing Medical University, No. 300 Guangzhou Road, Nanjing 210029, China; Department of Cardiology, The First Affiliated Hospital, Nanjing Medical University, No. 300 Guangzhou Road, Nanjing 210029, China; Cardiac Arrhythmia Center, Fuwai Hospital, National Center for Cardiovascular Diseases, Chinese Academy of Medical Sciences and Peking Union Medical College, No. 167 Beilishi Road, Beijing 100037, China; Department of Cardiology, The First Affiliated Hospital, Nanjing Medical University, No. 300 Guangzhou Road, Nanjing 210029, China

**Keywords:** Heart failure treatment, Left bundle branch area pacing, Left ventricular septal pacing, Left ventricular pacing, Cardiac resynchronization therapy

## Abstract

**Aims:**

Left bundle branch pacing is effective for cardiac resynchronization therapy (CRT), but the role of left ventricular septal pacing (LVSP) for CRT remains controversial due to lack of LBB capture. We hypothesized that combining LVSP with LV pacing (LVP) may provide additional benefits.

**Methods and results:**

This prospective observational study enrolled consecutive patients undergoing LVSP for CRT. LVSP was acceptable if paced QRS duration (QRSd)＜130 ms or QRSd reduction ≥ 20%. If neither criterion were met, a CS-LV lead was implanted. Acute hemodynamic response (AHR) represented by LV maximum first derivative (dP/dt_max_) was accessed. All patients were followed up for echocardiographic parameters, NT-proBNP levels, NYHA classes, and clinical events. The clinical outcomes included all-cause mortality, heart failure hospitalization, and ventricular tachyarrhythmias. A total of 45 patients achieved left bundle branch area pacing (LBBAP) without confirmed LBB capture were enrolled, including 25 with LVSP alone and 20 with LVSP + LVP. QRSd reduction was significantly greater in LVSP + LVP than LVSP (46.2 ± 19.2 ms vs. 32.6 ± 23.0 ms, *P* = 0.049). LVSP + LVP resulted in greater improvement in AHR than LVSP (20.0 ± 9.2% vs. 10.4 ± 8.2%, *P*＜0.001) in 10 patients. After a median follow-up of 26-month, LVEF improvement was significantly higher in LVSP + LVP than LVSP (mean difference: 3.05%; 95% CI: 0.05–6.05; *P* = 0.047). LVSP + LVP was also independently associated with 87% lower risk of clinical outcomes compared with LVSP [aHR: 0.13 (0.03, 0.62), *P* = 0.011].

**Conclusion:**

LVSP combined with LVP might offer greater AHR, electrical resynchronization and as well as improved clinical outcomes than LVSP alone in patients undergoing LBBAP-CRT without LBB capture.

## Graphical Abstract

**Graphical Abstract:**
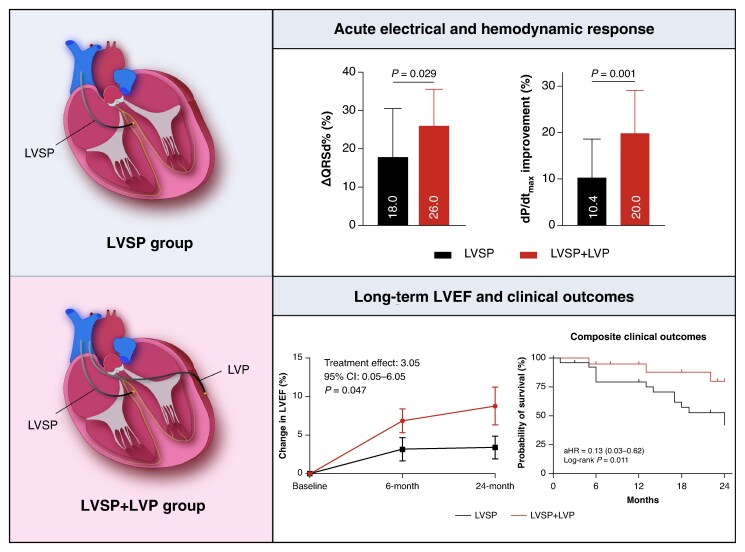

What’s New?

- This study demonstrated that in HF patients unable to achieve left bundle branch block correction with left bundle branch area pacing (LBBAP), left ventricular septal pacing (LVSP) combined with CS-LV lead implantation provided enhanced electrical resynchronization, improved acute hemodynamic response, and reduced incidence of clinical outcomes compared with LVSP only pacing modality.
- Additional CS-LV lead implantation can be considered in patients undergoing LBBAP-CRT to improve resynchrony and HF clinical outcomes.

## Introduction

Introduced recently in clinical practice, left bundle branch area pacing (LBBAP) has been demonstrated to be effective for heart failure (HF), particularly in patients with left bundle branch block (LBBB).^1^ Compared with conventional biventricular pacing (BiVP), LBBAP has the potential to achieve better echocardiographic resynchronization^1,2^ and hemodynamic benefit.^3^ Based on whether left bundle branch (LBB) is captured, LBBAP can be further classified into true left bundle branch pacing (LBBP) and left ventricular septal pacing (LVSP).^4^ Studies have suggested that LBBP may offer superior left ventricular synchrony compared with LVSP, as the LBBP involves faster activation of left ventricle through the conduction system.^5,6^ Recently, both our study and that of Diaz *et al*. have demonstrated that LBBP is associated with better long-term clinical outcomes compared with LVSP.^7,8^ In clinical practice, however, ∼21.5% of patients undergoing LBBAP did not achieve confirmed LBB capture.^9^ In HF patients with advanced cardiomyopathy, the likelihood of LBBAP without LBB capture might be particularly pronounced because intraventricular conduction delay (IVCD) or coexisting His-Purkinje system disease complicates achieving LBB capture. LVSP is sometimes accepted due to its effect for shortening QRS duration (QRSd) and potential benefit to reduce the risk of HF rehospitalization events. Previous studies have suggested that combination of conduction system pacing (CSP) with epicardial left ventricular pacing (LVP) via an additional coronary sinus (CS) LV lead may enhance electrical resynchronization.^10,11^ Chen *et al*. showed that LBBAP combining with LVP (LOT-CRT) provided greater ventricular electrical synchrony and clinical outcomes as compared with BiVP in patients with IVCD.^12^ However, the comparisons of effectiveness between LVSP and its combination with LVP for CRT remains limited. We hypothesized that combining LVSP with LVP could yield greater clinical benefits than LVSP alone. The study aimed to investigate the difference in acute hemodynamic responses (AHR), electrical resynchrony and clinical outcomes between LVSP with and without additional LVP.

## Methods

### Study patients

This was a two-centre prospective, observational study in First Affiliated Hospital of Nanjing Medical University and Fuwai hospital. Patients underwent LBBAP for CRT between January 2018 and December 2024 were screened. All patients had symptomatic HF and indications for CRT: Aged >18 years with wide QRSd ≥150 ms, reduced left ventricular ejection fraction (LVEF ≤ 40%), and NYHA class II-IV. Patients were included if LVSP was identified due to lacking evidence of LBB capture. LVSP alone was acceptable if paced QRSd was <130 ms or QRSd was reduced by ≥20% following LBBAP. An additional CS-LV lead was attempted when paced QRSd ≥130 ms or QRSd reduction < 20%. The exclusion criteria included: (i) patients undergoing CRT or upgrade with existing lead; (ii) patients undergoing successful LBBP with evidence of LBB capture; (iii) patients with persistent atrial fibrillation undergoing AV node ablation; (iv) patients with incomplete data. From June 2023 to November 2024, the patients in First Affiliated Hospital of Nanjing Medical University were consecutively screened and underwent hemodynamic evaluation during procedure to compare the difference between the two pacing strategies of LVSP alone and LVSP + LVP. Patients were excluded for frequent pre-mature ventricular complexes, moderate-to-severe aortic stenosis, LV thrombus, or inability to endure the procedure duration. The Review Board of two hospitals approved the study protocol and all patients provided written informed consent.

### Implanting procedure

LBBAP was performed using a Select Secure pacing lead (model 3830, 69 cm, Medtronic Inc., Minneapolis, MN) and a fixed-curve sheath (C315 HIS, Medtronic Inc., Minneapolis, MN), following established protocols.^4,13,14^ Briefly, the pacing lead was introduced into the right ventricle (RV) and was advanced from the right side of the interventricular septum (IVS) to the left side. A paced RBBB or QS morphology was observed in electrocardiogram (ECG) lead V1 when the lead reached the deep septum. Continuous monitoring with a 12-lead surface ECG, intracardiac electrograms, and fluoroscopy was performed throughout the procedure. Pacing stimulus to LV activation time (LVAT) in lead V5 or V6 was measured at low and high outputs. The lead tip was considered to be at the final position once LBBAP was identified successful. LVSP was defined by a RBBB or QS pattern in lead V1 during unipolar pacing, the lack of transition from non-selective to selective LBBP or from non-selective to LVSP, the lack of abrupt change of LVAT more than 10 ms during testing at different output and fulfilling at least one of the following criteria: V1–V6 interpeak interval ≤33 ms; difference of LVAT between HBP and LBBAP ≤9 ms. Patients with confirmed evidence of LBB capture were excluded. LVSP was accepted for CRT if paced QRSd was <130 ms or QRSd reduction ≥20%. An additional CS-LV lead would be attempted when paced QRSd ≥130 ms or QRSd reduction <20%. If the CS-LV lead was successfully implanted to the posterolateral or lateral branches, LVSP combined with LVP was selected for CRT. If CS-LV lead implantation failed, LVSP alone was accepted.

### Acute hemodynamic response

After successful implantation of all pacing leads, a pressure micromanometer (Pressure Wire X, Abbott, St. Paul, Minnesota) was placed in the LV cavity via retrograde transaortic catheterization through the radial artery, and subsequently connected to a pressure recording system (Quantine, Abbott, St. Paul, Minnesota). To mitigate the risk of developing thromboembolic complications, a bolus of intravenous unfractionated heparin was administered. Initially, hemodynamic data were acquired in AAI mode for 10 to 15 s as a control, then *trans* to DDD mode for another 10 to 15 s, employing either LVSP or LVSP + LVP modality. A total of eight transitions between AAI and DDD modes were accomplished with a fixed rate of 10 beats per minute above the patient’s intrinsic heart rate and an AV delay of 100 ms. LV pressure maximal first derivative (dP/dt_max_) was calculated using custom software (MATLAB R2023b; MathWorks, Inc.).The analysis excluded beats with ventricular or supraventricular extrasystoles and the subsequent beats. The percentage change in dP/dt_max_ from AAI to DDD or DDD to AAI was calculated for each transition.

### Data collection and follow-up

Demographic data, comorbidities, ECG and echocardiographic parameters, medications, and clinical status were collected. Procedural and fluoroscopy time for both LBBAP and CS-LV lead implantation were recorded. Follow-up visits occurred at 1−, 3−, and 6-months post-procedure, and annually thereafter. During each visit, pacing parameters including R-wave amplitudes, capture threshold, lead impedance, pacing percentage, and surface 12-lead ECG at the programmed voltage were documented. QRSd for both intrinsic and paced rhythms was measured from the earliest onset to the latest offset of QRS wave. LVAT was measured from the pacing stimulus to R-wave peak in lead V5 or V6. Echocardiograms were performed pre-procedure and every 6 months post-procedure to assess left ventricular function by experienced specialists. Clinical status was evaluated at baseline and during follow-up according to New York Heart Association (NYHA) class and NT-proBNP level.

### Evaluation of cardiac function and clinical outcomes

The improvement in LVEF was compared between baseline and last follow-up period. An echocardiographic response was defined as an increase in LVEF of ≥5%, while a super response was defined as an increase of ≥15%. The clinical outcomes included all-cause mortality, HF hospitalization (HFH) and ventricular tachyarrhythmias (VTAs). VTAs were defined as sustained ventricular tachycardia, ventricular fibrillation/flutter, or appropriate implantable cardioverter-defibrillator shock.

### Statistical analysis

Continuous variables with a normal distribution were expressed as mean ± standard deviation (SD) and compared using Student’s *t*-test, while those with a non-normal distribution were reported as median (Q1-Q3) and compared using the Mann–Whitney *U* test. Categorical variables were presented as frequencies or percentages and analysed using the χ^2^ test or Fisher’s exact test when expected cell counts were <5. Kaplan–Meier survival curves and log-rank tests were employed to compare the time to the first event between the LVSP and LVSP + LVP groups. Hazard ratios (HR) and 95% confidence intervals (CI) for outcome risks were estimated using Cox proportional hazards models, adjusted for relevant covariates. A two-tailed *P*-value of <0.05 was considered statistically significant. All statistical analyses were conducted using SPSS version 21.0 (IBM Corp, Armonk, NY, USA).

## Results

### Baseline characteristics

**Figure 1:**
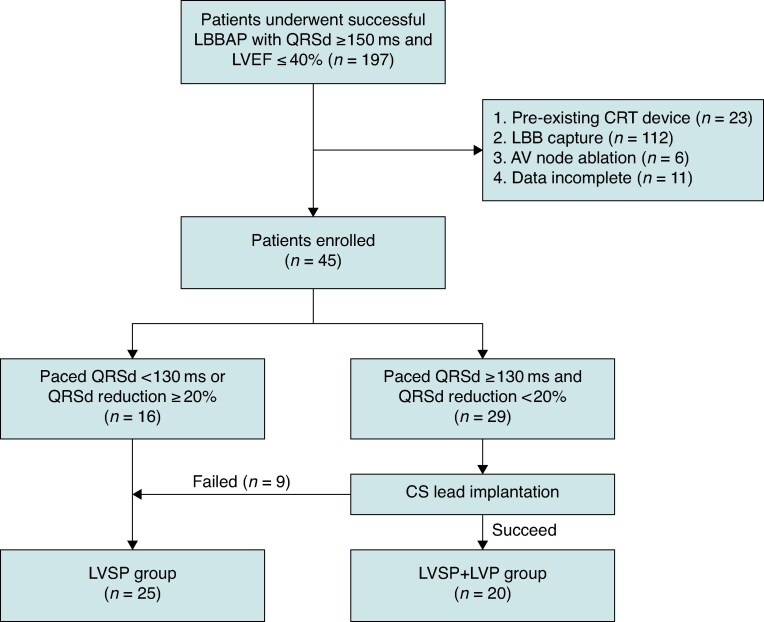
Flow chart of patients screening in the study. LBBAP, left bundle branch area pacing; QRSd, QRS duration; LVEF, left ventricular ejection fraction; CRT, cardiac resynchronization therapy; LBB, left bundle branch; AV, atrioventricular; CS, coronary sinus; LVSP, left ventricular septal pacing; LVP, epicardial left ventricular pacing.

Among 218 patients screened for LBBAP implantation, 197 patients achieved successful procedure. After excluding 23 patients with pre-existing CRT device or upgrade with existing lead, 112 patients with confirmed LBB capture, 6 patients with persistent atrial fibrillation undergoing AV node ablation, and 11 patients whose data were incomplete, eventually 45 patients were included in the study, with 25 in LVSP group and 20 in LVSP + LVP group. In the LVSP group, 16 patients had paced QRSd <130 ms or significant reduction of QRSd ≥20% (1 patient met the criterion of paced QRSd <130 ms, 12 patients met the criterion of ≥20% QRSd reduction and 3 patients met both of the criteria), and 9 were crossed over from the LVSP + LVP group due to CS-LV lead implantation failure (*Figure 1*). Baseline characteristics are summarized in *Table 1* and detailed in Supplementary material online, *Table S1*. The mean age was 62.1 ± 11.8 years, 34 (75.6%) were male. The intrinsic QRSd was 173.9 ± 18.4 ms, and the baseline LVEF was 30.8 ± 6.8%.

**Table 1:** Baseline characteristics of between LVSP and LVSP + LVP groups

	LVSP (*n* = 25)	LVSP + LVP (*n* = 20)	*P-*value
Demographics			
Male, *n*%	21 (84.0)	13 (65.0)	0.176
Age (years)	62.6 ± 11.5	61.5 ± 12.3	0.768
SBP (mmHg)	115.0 ± 17.7	110.6 ± 16.5	0.397
DBP (mmHg)	66.6 ± 9.6	70.5 ± 11.1	0.213
Comorbidities, *n* (%)			
NICM	18 (72.0)	19 (95.0)	0.059
Hypertension	6 (24.0)	9 (45.0)	0.205
Diabetes	5 (20.0)	7 (35.0)	0.320
CAD	10 (40.0)	3 (15.0)	0.100
CKD	2 (8.0)	1 (5)	>0.999
Atrial fibrillation	3 (12.0)	7 (35.0)	0.083
Electrocardiograph			
QRS duration (ms)	173.7 ± 19.2	174.1 ± 17.8	0.953
LBBB, *n*%	16 (64.0)	11 (55.0)	0.559
IVCD, *n*%	4 (16.0)	6 (30.0)	0.301
RBBB, *n*%	5 (20.0)	3 (15.0)	0.716
Echocardiogram			
LVEDD (mm)	68.7 ± 9.7	68.7 ± 13.0	0.993
LVESD (mm)	54.1 ± 5.9	58.9 ± 15.0	0.345
LVEF (%)	32.9 ± 6.7	28.2 ± 6.1	0.018
Medication, *n*%			
ACEI/ARB/ARNI	17 (68.0)	16 (80.0)	0.502
Beta blockers	23 (92.0)	19 (95.0)	>0.999
MRA	21 (84.0)	20 (100.0)	0.117
SGLT2i	7 (28.0)	9 (45.0)	0.348
Diuretic	21 (84.0)	19 (95.0)	0.362
Digoxin	8 (32.0)	7 (35.0)	>0.999
NYHA class, *n*%			
I	0 (0)	0 (0)	>0.999
II	9 (36.0)	5 (25.0)	0.526
III	12 (48.0)	11 (55.0)	0.767
IV	4 (16.0)	4 (20.0)	>0.999
NT-proBNP (pg/mL)	1591 [818.7, 3394]	1782 [478.8, 3582]	0.171

Data are presented as mean ± standard deviation for continuous variables and numbers and percentages for categorical variables.

LVSP, left ventricular septal pacing; LVP, epicardial left ventricular pacing; SBP, systolic blood pressure; DBP, diastolic blood pressure; NICM, non-ischemic cardiomyopathy; CAD, coronary heart disease; CKD, chronic kidney disease; LBBB, left bundle branch block; IVCD, intraventricular conduction delay; RBBB, right bundle branch block; LVEDD, left ventricular end-diastolic diameter; LVESD, left ventricular end-systole diameter; LVEF, left ventricular ejection fraction; ACEI/ARB/ARNI, angiotensin-converting enzyme inhibitor/angiotensin II receptor blocker/angiotensin receptor-neprilysin Inhibitor; MRA, aldosterone receptor antagonist; SGLT2i, sodium–glucose cotransporter-2 inhibitors; NYHA, New York heart association; RVP, right ventricular pacing; NT-proBNP, *n*-terminal pro-B-type natriuretic peptide.

### Acute electrical resynchronization and hemodynamic response

**Figure 2:**
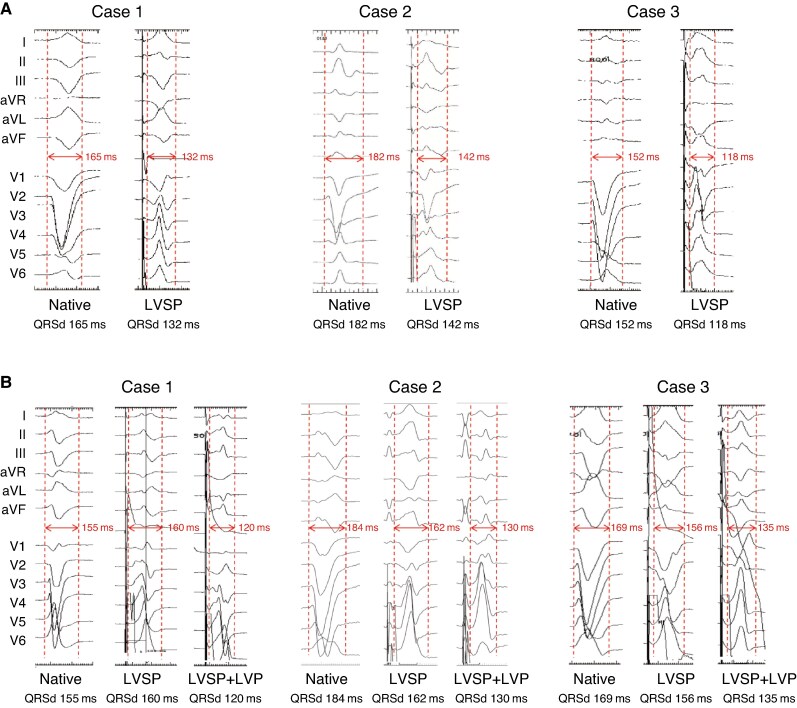
The examples of native and paced QRS in LVSP group and LVSP + LVP group. (*A*) Native and paced QRS during LVSP modality in LVSP group; (*B*) Native and paced QRS during LVSP and LVSP + LVP modality in LVSP + LVP group. LVSP, left ventricular septal pacing; LVP, epicardial left ventricular pacing.

**Figure 3:**
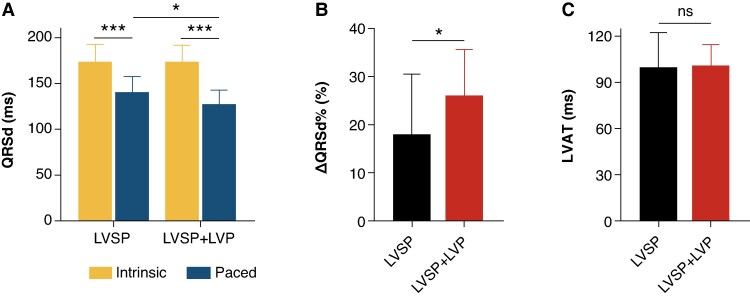
Acute electrical resynchronization in LVSP and LVSP + LVP. (*A*) QRS duration at intrinsic and paced rhythm in LVSP and LVSP + LVP group; (*B*) Proportion of QRS duration shortened compared with baseline in LVSP and LVSP + LVP; (*C*) Comparison of LVAT post-procedure in LVSP and LVSP + LVP. The data were the Mean ± SD. LVSP, left ventricular septal pacing; LVP, epicardial left ventricular pacing; LVAT, left ventricular activation time.

**Figure 4:**
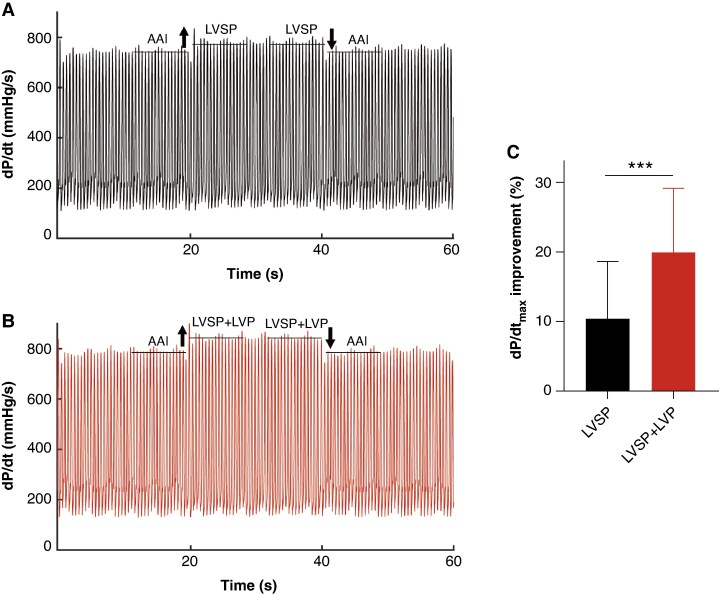
Comparison of dP/dt_max_ improvement during LVSP and LVSP + LVP. (*A*) The improvement of dP/dt_max_ from baseline (AAI mode) to LVSP in one patient was 16.7%; (*B*) The improvement of dP/dt_max_ from baseline (AAI mode) to LVSP + LVP was 29.0% in the same patient; (*C*) Comparison of dP/dt_max_ improvement during LVSP and LVSP + LVP. The data were the Mean ± SD. LVSP, left ventricular septal pacing; LVP, epicardial left ventricular pacing; AHR, acute hemodynamic response.

*Figure 2* shows 3 cases of native and paced QRS for LVSP group and LVSP + LVP group, respectively. There was no significant difference in baseline QRSd between LVSP and LVSP + LVP groups (173.7 ± 19.2 ms vs. 174.1 ± 17.8 ms, *P* = 0.953). As shown in *Figure 3*, LVAT were long and similar in both groups (LVSP: 99.8 ± 22.5 ms vs. LVSP + LVP: 101.1 ± 13.5 ms, *P* = 0.845). Paced QRSd was significantly reduced in both groups, with LVSP + LVP group achieving narrower paced QRSd compared with LVSP group (127.3 ± 15.7 ms vs. 140.5 ± 17.3 ms, *P* = 0.015). The reduction in QRSd was more pronounced in LVSP + LVP group (26.0 ± 9.6% vs. 18.0 ± 12.5%, *P* = 0.029). The mean dP/dt_max_ was 884.9 ± 300.6 mmHg/s at baseline. An example of the dP/dt improvement achieved by LVSP alone vs. LVSP + LVP is illustrated in *Figure 4A* and *B*. LVSP + LVP resulted in greater improvement in dP/dt_max_ in all 10 patients compared with LVSP alone (20.0 ± 9.2% vs. 10.4 ± 8.2%, *P* = 0.001) (*Figure 4C*).

### Improvement in cardiac function during follow-up

**Figure 5:**
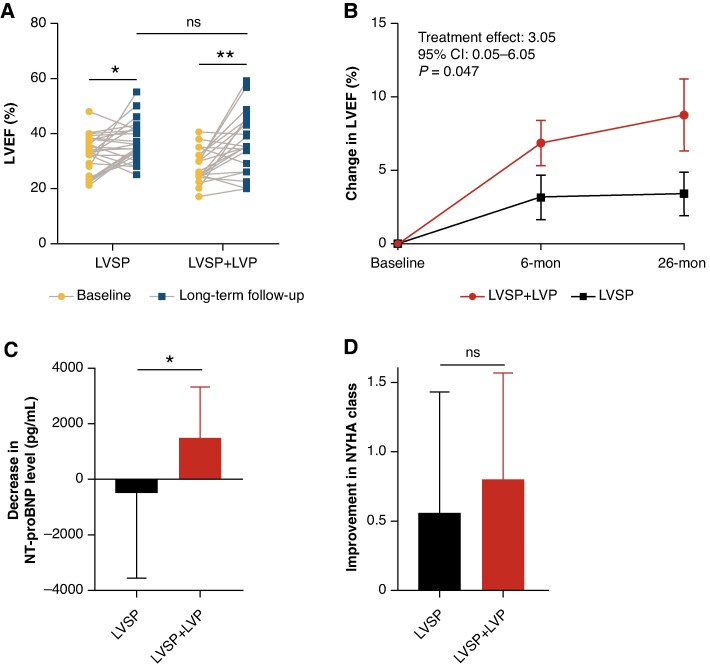
Change in cardiac function during long-term follow-up. (*A*) Comparison of LVEF from baseline to long-term follow-up in LVSP and LVSP + LVP; (*B*) The repeated measures mixed-effects model compared the improvement in LVEF from baseline between LVSP and LVSP + LVP; (*C*) Change of NT-proBNP levels in LVSP and LVSP + LVP from baseline to long-term follow-up; (*D*) Change of NYHA class in LVSP and LVSP + LVP from baseline to long-term follow-up. The data were the mean ± SD. LVSP, left ventricular septal pacing; LVP, epicardial left ventricular pacing; LVEF, left ventricular ejection fraction; NT-proBNP, N-terminal pro-B-type natriuretic peptide; NYHA, New York heart association.

Baseline LVEF was lower in LVSP + LVP group compared with LVSP group (28.3 ± 6.1% vs. 32.9 ± 6.7%, *P* = 0.018), however baseline LVEF was not a significant predictor for clinical outcomes in univariate analysis (see Supplementary material online, *Table S2*). Long-term echocardiographic parameters are presented in *Table 2* and *Figure 5*. In both LVSP and LVSP + LVP group, LVEF improved (LVSP: 32.9 ± 6.7% to 37.0 ± 7.1%, *P* = 0.019, LVSP + LVP: 28.3 ± 6.1% to 36.9 ± 11.3%, *P* = 0.002) from baseline. The results of the repeated measures mixed-effects model indicated that after adjusting for baseline LVEF levels, LVSP + LVP group showed a 3.05% greater improvement in LVEF compared with the LVSP group (95% CI: 0.05–6.05%, *P* = 0.047). Consistent with LVEF changes, left ventricular end-diastolic diameter (LVEDD) was reduced from 68.7 ± 9.7 mm to 64.6 ± 9.4 mm (*P* = 0.004) in LVSP group and from 68.7 ± 13.0 mm to 63.8 ± 12.9 mm (*P* = 0.005) in LVSP + LVP group. A total of 19 patients were classified as responders, with 8 in LVSP group and 11 in LVSP + LVP group (32.0% vs. 55.0%, *P* = 0.142) during the median 26-month follow-up. One patient (4%) in LVSP group and 6 (30.0%) in LVSP + LVP group met the criteria for super-response (*P* = 0.034).

**Table 2:** Comparison of echocardiographic data and clinical cardiac function of the LVSP group and the LVSP + LVP group

	LVSP (*n* = 25)	LVSP + LVP (*n* = 20)	*P-*value
Echocardiographic parameters			
Baseline LVEF (%)	32.9 ± 6.7	28.3 ± 6.1	0.018
6-month LVEF (%)	36.1 ± 6.6	35.1 ± 9.6	0.670
Change in LVEF at 6-month (%)	3.5 ± 7.3	6.9 ± 6.9	0.120
Long-term LVEF (%)	37.0 ± 7.1	36.9 ± 11.3	0.971
Change in LVEF at long-term (%)	4.1 ± 8.2	8.8 ± 11	0.112
Baseline LVEDD (mm)	68.7 ± 9.7	68.7 ± 13.0	0.993
Long-term LVEDD (mm)	64.6 ± 9.4	63.8 ± 12.9	0.808
Response (*n*, %)	8 (32.0)	11 (55.0)	0.142
Super-response (*n*, %)	1 (4)	6 (30.0)	0.034
Long-term clinical cardiac function			
Change in NT-proBNP, pg/mL	105.2 (−1017, 1010)	1399 (96.4, 2617)	0.030
Change in NYHA class	0.6 ± 0.9	0.8 ± 0.8	0.536

LVSP, left ventricular septal pacing; LVP, epicardial left ventricular pacing; LVAT, left ventricular activation time; LVEDD, left ventricular end-diastolic diameter; LVEF, left ventricular ejection fraction; NT-proBNP, *n*-terminal pro-B-type natriuretic peptide; NYHA, New York heart association.

NT-proBNP levels were similar between the groups at baseline (1591 [818.7, 3394] vs. 1782 [478.8, 3582], *P* = 0.171). However, after long-term follow-up, LVSP + LVP group showed a significantly greater decrease in NT-proBNP levels (1399 [96.38, 2617] vs. 105.2 [−1017, 1010] pg/mL, *P* = 0.030). NYHA class improved in both groups compared with baseline, though no significant difference was observed between the groups at long-term follow-up (*P* = 0.536) (*Table 2* and *Figure 5*).

### Improvement in clinical outcomes

**Figure 6:**
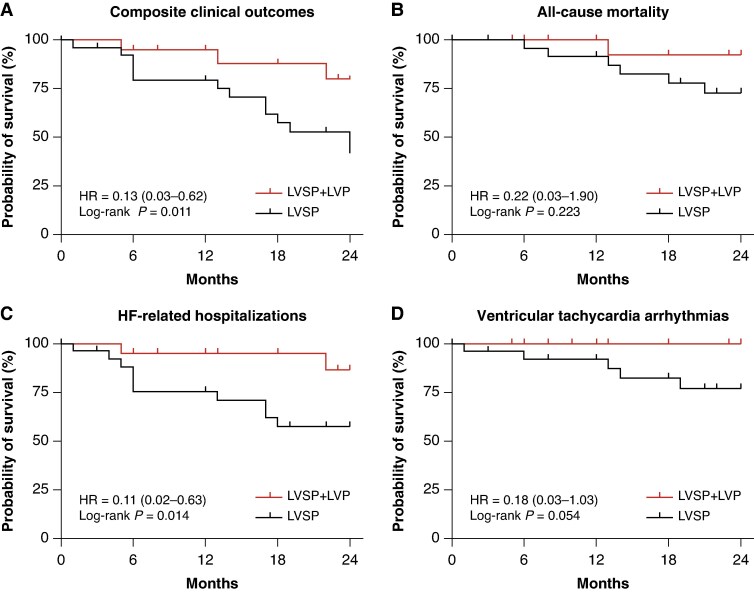
Clinical outcomes in LVSP and LVSP + LVP during long-term follow-up. (*A*) Comparison of composite clinical outcomes in LVSP and LVSP + LVP during long-term follow-up; (*B*) Comparison of all-cause mortality in LVSP and LVSP + LVP during long-term follow-up; (*C*) Comparison of HFH rate in LVSP and LVSP + LVP during long-term follow-up; (*D*) Comparison of VTAs rate in LVSP and LVSP + LVP during long-term follow-up. LVSP, left ventricular septal pacing; LVP, epicardial left ventricular pacing; HF, heart failure.

Clinical outcomes occurred in 16 patients (13 in the LVSP group vs. 3 in the LVSP + LVP group, *P* = 0.013). Multivariate Cox regression models were applied to assess the long-term outcomes between LVSP and LVSP + LVP. After adjusting for baseline LVEF and other variables with a *P*-value of <0.05 in univariate analysis (see Supplementary material online, *Table S2*), LVSP + LVP was independently associated with 87% lower risk of clinical outcomes compared with LVSP [aHR: 0.13 (0.03, 0.62), *P* = 0.011] during long-term follow-up. LVSP + LVP was also linked to a significantly lower incidence of HFH (10.0% vs. 40.0%, *P* = 0.040), and a lower trend in incidence of all-cause mortality (5.0% vs. 24.0%, *P* = 0.117) and ventricular tachyarrhythmias (VTAs) (0% vs. 20.0%, *P* = 0.056) (*Figure 6*).

## Discussion

The main findings of this study are as follows: In HF patients unable to achieve LBB capture during LBBAP-CRT, (i) LVSP combined with LVP achieves better acute electrical resynchronization and hemodynamic response than LVSP alone; (ii) LVSP + LVP had greater improvement in LVEF and more significant reduction in NT-proBNP levels; (iii) compared with LVSP, LVSP + LVP was associated with a lower incidence of composite clinical endpoints, particularly significantly lower rate of HFH during long-term follow-up.

### LVSP in heart failure patients

LBBAP is the preferred CSP strategy,^15,16^ but there is growing concern about LBB capture failure. The European MELOS study reported 21.5% LVSP incidence among LBBAP patients.^9^ In the real-world clinical practice, HF patients undergoing LBBAP are more likely to achieve LVSP. Notably, > 50% of our cohort exhibited baseline ECG of LBBB, but LBB capture could not be achieved. We found that their LVEDD was significantly enlarged with a mean value of 68.7 mm, indicating more severe and advanced cardiomyopathy. And Graterol *et al*. reported LVEDD＞60 mm predicts a failed LBBP implant (60% sensitivity/71% specificity).^17^ Computational modelling has demonstrated that LVSP performs at least similar to BiVP in terms of hemodynamics in structurally normal hearts.^18^ Furthermore, long-term outcomes between LBBP, LVSP, and BiVP in HF patients indicated that LVSP is less effective than LBBP and is comparable to BiVP in LVEF and functional improvements.^7,8^ In the real-world practice, LVSP is acceptable when paced QRSd is <130 ms or reduced ≥20%. However, when paced QRSd is suboptimal, the combination of LVSP with LVP, as shown in our study, may offer a viable alternative for CRT strategy for patients who LBB capture cannot be achieved.

### Resynchronization pacing strategies for LVSP in heart failure patients

In HF patients with combined proximal His-Purkinje conduction block and diffuse distal myocardial pathology, CSP may inadequately correct ventricular dyssynchrony.^19^ Furthermore, a prospective study revealed that 36% of patients with ECG-defined LBBB exhibit complete Purkinje activation, a substrate wherein intrinsic Purkinje disease prevents QRS narrowing even with successful His-bundle capture.^20^ An innovative procedure of combining LV epicardial pacing in LBBAP has been proposed safe and feasible and offers advantage over BiVP and LBBAP for resynchronization in HF patients.^10^ A study focused on IVCD patients showed that LOT-CRT has better results in electrical and echocardiographic improvement and clinical response than BiVP.^12^

Previous studies have indicated that both endocardial LVSP and transeptal LVSP are comparable to BiVP in view of acute hemodynamic mechanism.^21,22^ The benefits of combining LVP with LBBAP was proved in the recent CSPOT study.^23^ The primary results demonstrated that LOT-CRT provided improved maximum rate of pressure rise in LV compared with LBBAP alone. In the CSPOT study, LBBAP actually included some true LBBP and some LVSP, while in our study, only patients diagnosed with confirmed LVSP were included, although 22.2% (10/45) of them did not achieve terminal R in lead V1. The present study demonstrated that LVSP + LVP provided superior electrical resynchronization compared with LVSP alone. Notably, in self-controlled comparisons, acute hemodynamic improvement was also significantly better in LVSP + LVP modality than in LVSP alone. The results suggest that combining LVSP with LVP provides additional benefits, which is consistent with the results of the CSPOT study, where combining LVP with LBBAP was most effective in patients with deep septal pacing.^23^ Our LVSP cohort demonstrated modest LVEF improvement, likely attributable to failure to capture the conduction system. Combining LVSP with LVP had more LVEF improvement and more significant LVEDD reduction during long-term follow-up, in line with the improvement in electrical resynchronization, suggesting that improved electrical resynchronization has the possibility of bringing about improved mechanical resynchronization to some extent. NT-proBNP level decreased significantly in patients with LVSP + LVP, however no statistics difference in change in LVSP from baseline. For clinical outcomes, LVSP + LVP group was associated with a significantly lower risk than LVSP alone, especially for risk of HFH. The present study demonstrated the advantages of combined CS-LV lead implantation with LVSP in the LBBAP procedure. On the contrary, there is also a clinical dilemma regarding the need to implant additional LBBAP leads when using conventional BiVP as the initial strategy for CRT. A recent study indicates that BiVP combined with LBBAP (LOT-CRT) had a better outcome compared with BiVP when a QLV ratio (QLV/baseline QRS value) below 70%.^24^ Therefore, the combination of LBBAP and LVP may be pursued in cases with suboptimal paced QRSd as suggested in the latest ESC consensus.^25^

## Limitations

First, this study is limited by its relatively small sample size. Future studies with larger populations are needed to confirm these findings. The prognosis for patients with LVSP is not promising due to the inability to achieve true cardiac resynchronization therapy without LBB capture. To date, few studies have specifically examined the potential benefits of adding a CS-LV lead in patients with LVSP. Our study demonstrated that combining LVSP with LVP is superior to LVSP alone in terms of improvements in electrical and hemodynamic effects, as well as clinical outcomes. Second, only limited self-controlled acute hemodynamic comparison were reported in our study due to unavailability of pressure wires in the first half of this study that introduces bias to our results. Third, there were nine patients crossed over to LVSP only group due to failure of CS-LV lead placement, actually limiting the value of two groups’ comparison. Fourth, there was no direct comparison with conventional BiVP in this study, leaving a gap in assessing the efficacy of LVP alone for patients without confirmed LBB capture. However, previous research has indicated that BiVP is less effective than combining LBBAP with LVP in patients with IVCD.^12^ Fifth, in our study, LVSP was defined only by excluding confirmed LBBP. We did not perform programmed stimulation during our procedure, so, we cannot completely exclude LBBP in LVSP cases. Finally, we measured LVAT in lead V5 when R-waves appeared notched in V6, which resulted in slightly shorter LVAT in V5 compared with V6, also shown in other reported measurements.^26^

## Conclusions

LVSP combined with LVP was associated with greater improvement in electrical resynchronization and AHR, and lower incidence of clinical outcomes during long-term follow-up. Additional CS-LV lead implantation is recommended for LVSP. Large-scale trials are warranted to further validate these findings.

## Supplementary Material

euaf147_Supplementary_Data

## Data Availability

The data underlying this article will be shared on reasonable request to the corresponding author.
