# Supplementary material for: Left ventricular septal pacing combined with left ventricular pacing improves acute electric resynchronization, hemodynamic responses and clinical outcomes: results of SPORT study
Source: Europace. 2025 Aug 4;27(8):euaf147. doi: 10.1093/europace/euaf147 (PMC12319671; doi:10.1093/europace/euaf147)
Supplement: euaf147_Supplementary_Data [file euaf147_supplementary_data.docx]

**Supplementary Material**

**Supplementary Table S1. Baseline patient demographics**

| **Patient** | **Gender** | **Age(y)** | **LVEF(%)** | **Etiology** | **Rhythm** | **QRS morphology** | **QRSd (ms)** |
| --- | --- | --- | --- | --- | --- | --- | --- |
| LVSP 1 | M | 69 | 35 | ICM | SR | IVCD | 171 |
| LVSP 2 | M | 68 | 36 | NICM | SR | RBBB | 162 |
| LVSP 3 | F | 70 | 33 | NICM | SR | IVCD | 152 |
| LVSP 4 | M | 73 | 32 | NICM | SR | LBBB | 218 |
| LVSP 5 | M | 57 | 38 | NICM | SR | RBBB | 182 |
| LVSP 6 | M | 78 | 29 | ICM | SR | IVCD | 170 |
| LVSP 7 | M | 80 | 34 | NICM | SR | LBBB | 173 |
| LVSP 8 | M | 73 | 32 | ICM | AF | LBBB | 151 |
| LVSP 9 | F | 55 | 36 | NICM | SR | LBBB | 165 |
| LVSP 10 | M | 74 | 38 | ICM | SR | RBBB | 213 |
| LVSP 11 | M | 55 | 22 | NICM | SR | LBBB | 162 |
| LVSP 12 | M | 61 | 21 | NICM | SR | LBBB | 153 |
| LVSP 13 | M | 47 | 23 | NICM | SR | LBBB | 204 |
| LVSP 14 | F | 70 | 32 | NICM | SR | LBBB | 178 |
| LVSP 15 | M | 49 | 33 | NICM | SR | IVCD | 150 |
| LVSP 16 | M | 29 | 23 | NICM | AF | LBBB | 179 |
| LVSP 17 | M | 54 | 37 | NICM | SR | LBBB | 156 |
| LVSP 18 | M | 49 | 28 | NICM | SR | LBBB | 196 |
| LVSP 19 | M | 67 | 32 | NICM | SR | LBBB | 186 |
| LVSP 20 | M | 57 | 39 | ICM | SR | RBBB | 178 |
| LVSP 21 | M | 66 | 40 | NICM | SR | LBBB | 178 |
| LVSP 22 | M | 60 | 38 | ICM | SR | LBBB | 183 |
| LVSP 23 | F | 71 | 40 | ICM | SR | LBBB | 152 |
| LVSP 24 | M | 65 | 48 | NICM | AF | RBBB | 153 |
| LVSP 25 | M | 67 | 24 | NICM | SR | LBBB | 178 |
| LVSP+LVP 1 | M | 46 | 17 | NICM | SR | LBBB | 219 |
| LVSP+LVP 2 | M | 56 | 22 | NICM | SR | IVCD | 187 |
| LVSP+LVP 3 | F | 55 | 24 | NICM | SR | LBBB | 193 |
| LVSP+LVP 4 | M | 67 | 24 | NICM | SR | LBBB | 182 |
| LVSP+LVP 5 | M | 54 | 40 | NICM | SR | IVCD | 156 |
| LVSP+LVP 6 | M | 73 | 32 | NICM | AF | LBBB | 183 |
| LVSP+LVP 7 | M | 58 | 35 | NICM | AF | RBBB | 184 |
| LVSP+LVP 8 | M | 76 | 32 | ICM | AF | LBBB | 169 |
| LVSP+LVP 9 | M | 74 | 36 | NICM | AF | IVCD | 160 |
| LVSP+LVP 10 | F | 71 | 25 | NICM | SR | IVCD | 155 |
| LVSP+LVP 11 | F | 67 | 24 | NICM | SR | LBBB | 167 |
| LVSP+LVP 12 | M | 77 | 30 | NICM | AF | LBBB | 183 |
| LVSP+LVP 13 | F | 54 | 26 | NICM | SR | IVCD | 180 |
| LVSP+LVP 14 | M | 49 | 24 | NICM | SR | RBBB | 182 |
| LVSP+LVP 15 | F | 36 | 38 | NICM | AF | RBBB | 153 |
| LVSP+LVP 16 | M | 50 | 30 | NICM | SR | LBBB | 160 |
| LVSP+LVP 17 | M | 66 | 30 | NICM | SR | LBBB | 172 |
| LVSP+LVP 18 | M | 49 | 20 | NICM | SR | IVCD | 150 |
| LVSP+LVP 19 | F | 79 | 25 | NICM | AF | LBBB | 152 |
| LVSP+LVP 20 | F | 73 | 30 | NICM | SR | LBBB | 194 |

LVSP = left ventricular septal pacing; LVP = epicardial left ventricular pacing; LVEF = left ventricular ejection fraction; NICM = non-ischemic cardiomyopathy; SR = sinus rhythm; AF = atrial fibrillation; LBBB=left bundle branch block; IVCD = intraventricular conduction delay; RBBB = right bundle branch block

**Supplementary Table S2. Cox regression analysis assessing the relationship between characteristics and clinical outcomes**

|  | **Clinical outcomes** | | **All-cause Mortality** | | **HF Hospitalization** | | **Ventricular Tachycardia Arrhythmias** | |
| --- | --- | --- | --- | --- | --- | --- | --- | --- |
|  | HR(95% CI) | *P*  value | HR(95% CI) | *P*  value | HR(95% CI) | *P*  value | HR(95% CI) | *P*  value |
| LVSP+LVP vs. LVSP Group | ***0.179***  ***(0.039-0.819)*** | ***0.027*** | 0.241  (0.029-1.999) | 0.187 | ***0.103***  ***(0.013-0.822)*** | ***0.032*** | 0.020  (0.001-9.953) | 0.294 |
| Gender | 1.088  (0.298-3.972) | 0.899 | 0.525  (0.063-4.366) | 0.551 | 2.183  (0.691-6.900) | 0.184 | 0.032  (0.003-3,409) | 0.436 |
| Age | 1.017  (0.964-1.072) | 0.543 | 0.992  (0.927-1.062) | 0.819 | 1.020  (0.966-1.078) | 0.470 | 0.959  (0.892-1.032) | 0.264 |
| SBP | ***0.949***  ***(0.911-0.989)*** | ***0.013*** | 0.963  (0.915-1.015) | 0.158 | 0.971  (0.936-1.008) | 0.129 | 0.975  (0.920-1.033) | 0.397 |
| DBP | 0.949  (0.899-1.002) | 0.060 | 0.932  (0.862-1.009) | 0.082 | 0.957  (0.905-1.013) | 0.130 | 0.968  (0.888-1.056) | 0.465 |
| NICM | 0.694  (0.191-2.527) | 0.580 | 1.351  (0.163-11.224) | 0.781 | 0.626  (0.169-2.313) | 0.482 | 0.830  (0.093-7.439) | 0.868 |
| Hypertension | 0.700  (0.193-2.546) | 0.588 | 0.893  (0.173-4.612) | 0.893 | 0.684  (0.185-2.526) | 0.568 | 0.573  (0.064-5.140) | 0.619 |
| Diabetes | 1.713  (0.525-5.589) | 0.372 | 1.407  (0.273-7.260) | 0.683 | 2.598  (0.082-8.234) | 0.105 | 0.863  (0.096-7.752) | 0.895 |
| CAD | 1.092  (0.336-3.552) | 0.883 | 0.974  (0.189-5.021) | 0.974 | 1.775  (0.563-5.595) | 0.327 | 1.665  (0.278-9.972) | 0.577 |
| CKD | 0.867  (0.113-6.678) | 0.891 | 0.043  (0.007-7.752) | 0.597 | 0.880  (0.113-6.825) | 0.902 | 1.370  (0.010-13.015) | 0.840 |
| Atrial fibrillation | 1.080  (0.297-3.930) | 0.907 | 1.559  (0.302-8.039) | 0.596 | 0.675  (0.148-3.080) | 0.612 | 0.917  (0.102-8.214) | 0.938 |
| QRS duration | 0.992  (0.966-1.018) | 0.535 | 1.007  (0.975-1.041) | 0.661 | 0.991  (0.963-1.019) | 0.523 | 0.988  (0.946-1.032) | 0.581 |
| LBBB | 1.179  (0.385-3.609) | 0.773 | 1.928  (0.373-9.959) | 0.433 | 0.959  (0.304-3.026) | 0.943 | 1.098  (0.183-6.594) | 0.918 |
| LVEF | 0.987  (0.908-1.073) | 0.761 | 0.993  (0.887-1.111) | 0.897 | 1.018  (0.937-1.106) | 0.678 | 0.953  (0.832-1.091) | 0.485 |
| ACEI/ARB/ARNI | 0.667  (0.218-2.039) | 0.477 | 2.523  (0.304-20.963) | 0.392 | 2.181  (0.478-9.965) | 0.314 | 1.735  (0.193-15.569) | 0.622 |
| Beta blockers | 0.986  (0.128-7.599) | 0.989 | 1.172  (0.138-152.930) | 0.648 | 0.878  (0.113-6.808) | 0.901 | 0.885  (0.094-117.344) | 0.691 |
| MRA | 1.601  (0.208-12.346) | 0.651 | 2.398  (0.282-313.159) | 0.535 | 1.397  (0.749-10.854) | 0.749 | 1.597  (0.168-212.468) | 0.596 |
| SGLT2i | 1.436  (0.370-5.573) | 0.601 | 0.661  (0.078-5.613) | 0.705 | 1.462  (0.421-5.083) | 0.550 | 0.031  (0.003-4.274) | 0.451 |
| Diuretic | 1.594  (0.207-12.270) | 0.654 | 1.891  (0.223-246.593) | 0.513 | 0.679  (0.148-3.110) | 0.618 | 1.309  (0.139-173.544) | 0.582 |
| Digoxin | 0.352  (0.096-1.293) | 0.116 | 0.219  (0.026-1.827) | 0.161 | 0.129  (0.017-1.006) | 0.051 | 0.329  (0.036-2.991) | 0.323 |
| NT-proBNP | 1.000  (1.000-1.000) | 0.620 | 1.000  (0.999-1.000) | 0.485 | 1.000  (1.000-1.000) | 0.974 | 1.000  (1.000-1.000) | 0.735 |
| NYHA class | 1.270  (0.532-3.030) | 0.590 | 0.988  (0.314-3.104) | 0.984 | 1.277  (0.551-2.961) | 0.569 | 2.039  (0.545-7.629) | 0.290 |

SBP = systolic blood pressure; DBP = diastolic blood pressure; NICM = non-ischemic cardiomyopathy; CAD = coronary heart disease; CKD = chronic kidney disease; LBBB=left bundle branch block; LVEF = left ventricular ejection fraction; ACEI/ARB/ARNI = angiotensin-converting enzyme inhibitor/angiotensin II receptor blocker/angiotensin receptor-neprilysin Inhibitor; MRA = aldosterone receptor antagonist; SGLT2i = sodium-glucose cotransporter-2 inhibitorsNT-proBNP = N-terminal pro-B-type natriuretic peptide; NYHA = new york heart association; RVP = right ventricular pacing
